# Characterization of complete mitochondrial genome of *Polyura narcaeus* (Lepidoptera: Nymphalidae: Charaxinae)

**DOI:** 10.1080/23802359.2021.1927875

**Published:** 2021-05-13

**Authors:** Runyu Xu, Lizhong Duan, Xiangyu Hao, Jintian Xiao, Xiangqun Yuan

**Affiliations:** a College of Life Sciences, Northwest A&F University, Yangling, China; b Key Laboratory of Plant Protection Resources and Pest Management, Ministry of Education, Entomological Museum, College of Plant Protection, Northwest A&F University, Yangling, China

**Keywords:** Mitogenome, phylogeny, Nymphalidae, Charaxinae, *Polyura narcaeus*

## Abstract

The complete mitochondrial genome of *Polyura narcaeus* (Hewitson, 1854) (Lepidoptera: Nymphalidae: Charaxinae) was sequenced in the study. The circular genome is 15,319 bp in size and includes 13 protein-coding genes, 22 transfer RNA genes, 2 ribosomal RNA genes and a non-coding AT-rich region. The base composition of the whole mitogenome is 39.15% A, 42.08% T, 11.18% C and 7.59% G, showing a strong AT bias. The characteristics of encoding PCGs, rRNAs and tRNAs, as well as the non-coding intergenic spacers and overlapping sequences are nearly the same with other known butterflies. The AT-rich region also contains several features characteristic of the typical butterflies. Phylogenetic analysis distinctly showed that the family Nymphalidae was a monophyletic group, and that the newly determined *Polyura narcaeus* of this study was firstly sister to *Polyura nepenthes*, then they were clustered with *Polyura arja*.

Insect mitogenomes have been widely used in the studies of phylogeny, evolution and population genetics, etc (Galtier et al. 2009). *Polyura narcaeus* (Hewitson, 1854) is one representative species of the genus *Polyura* in the subfamily Charaxinae (Lepidoptera: Nymphalidae) which is commonly found in Palearctic and Indo-Malayan areas (Chou 1994; Seitz 1908). The adult individuals were collected at Huangshan Mountain (Anhui Province, China) (coordinates: E118°10′, N30°10′) in August 2018. After sample collection, the fresh tissues were immediately preserved in 100% ethanol and stored in the Entomological Museum of Northwest A&F University (URL: https://ppc.nwafu.edu.cn/english/aboutus/index.htm; Contact person: Xiangqun Yuan, yuanxq@nwsuaf.edu.cn) under the voucher number (NWAFU-20180504). The mitogenomic DNA was extracted from thoracic muscle using Genomic DNA Kit (TransGen Biotech, Beijing) and sequenced on an Illumina HiSeq 4000 platform (Novogene, Beijing). The genomic information referenced to *Polyura arja* (Wu et al. 2014) was assembled and annotated using Geneious 8.1.3.

The whole mitogenome (GenBank accession number: MW683125) is 15,319 bp in size and shows a strong AT bias (81.23%). The mitogenome totally contains 13 protein-coding genes (PCGs), 22 transfer RNA genes, 2 ribosomal RNA genes and an AT-rich region (control region). Thirteen of these 37 genes are encoded on major strand (J-strand) while other 24 are encoded on the minor strand (N-strand). The mitogenome has an identical gene order and orientation with other butterflies, and harbors 13 intergenic spacers ranging from 1 to 58 bp (145 bp in total) and 10 overlapping sequences from 1 to 8 bp (28 bp in total) scattered throughout the whole genome.

The 13 PCGs of the genome is 11,175 bp in total. All PCGs start with a typical ATN codon, except for the *cox1* which uses CGA as its start codons. Five of 13 PCGs stop with codon TAA and the others terminate with the single nucleotide T, which was also frequently found in most insect mitogenomes (Kim et al. 2011). The total length of 22 tRNAs is 1482 bp, ranging from 61 bp to 85 bp in size, which is also consistent with almost all other insect mitogeomes. All tRNA genes evidence the typical clover-leaf secondary structures except for *tRNA ^ser^*(AGN) which loses its dihydrouridine (DHU) arm. The *rrnL* and *rrnS* genes are 1,360 and 771 bp, with their AT contents of 84.8% and 85.6%, respectively. The 414 bp AT-rich region located between *rrnS* and *tRNA^Met^* harbors the highest A + T content (91.79%) of the whole genome. In addition, several short repeats of sequences characteristic of lepidopterans were scattered throughout the region: a 51 bp poly-T stretch, a microsatellite-like (AT)_9_ element as well as some single nucleotides repeats such as (T)_2–11_, (A)_2–7_.

The phylogenetic relationships of 33 nymphalid species availabe were reconstructed based on concatenated 13 PCG datasets with maximum likelihood (ML) method via PhyloSuite software, using two papilionid species as the outgroups. The result showed that the subfamily Charaxinae was recovered as a monophyletic group and that the newly determined *P. narcaeus* of this study was firstly sister to *P. nepenthes*, and then they clustered with *P. arja* (Figure 1).

**Figure 1.**
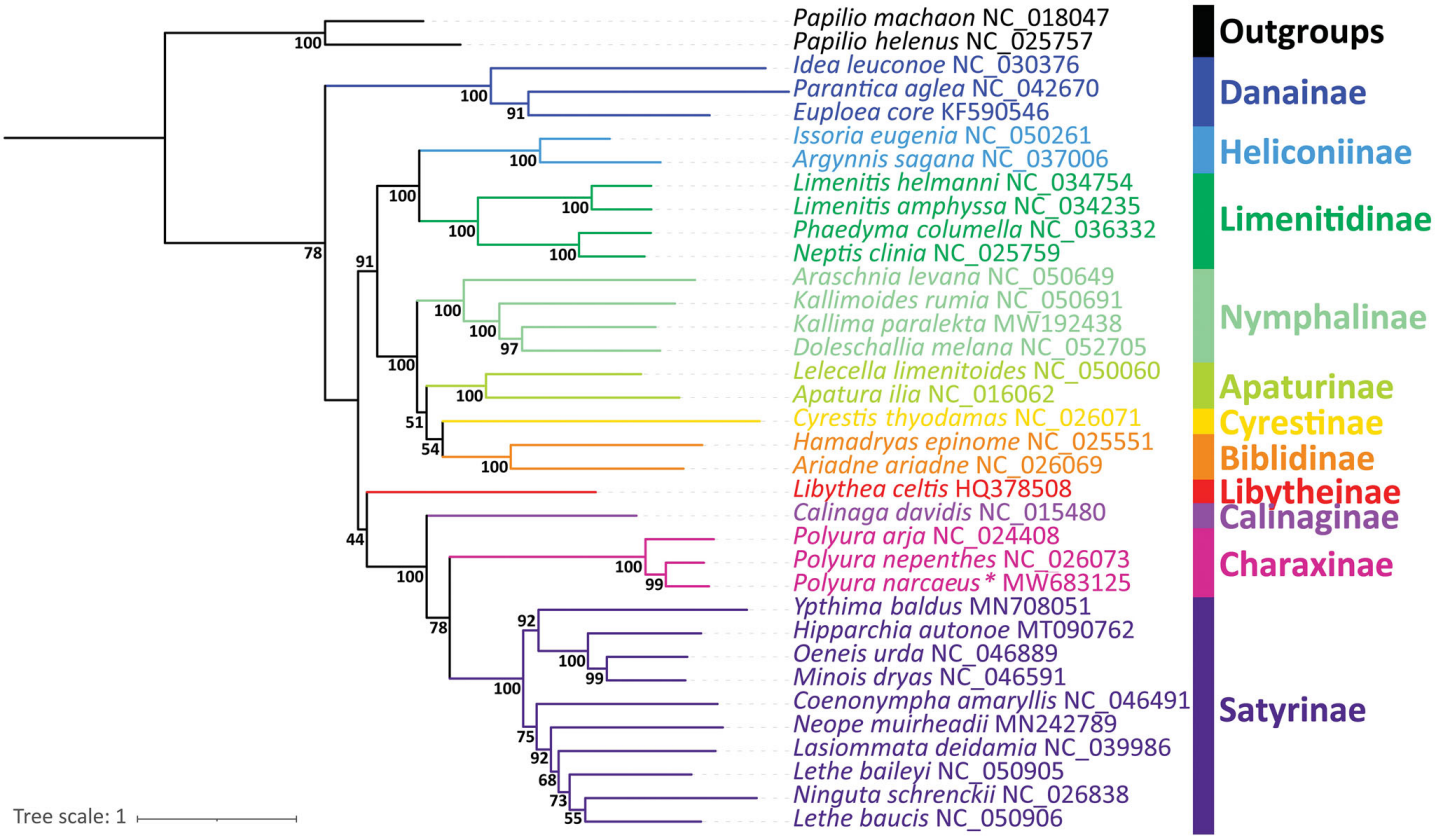
Phylogenetic relationships of Nymphalidae inferred from mitochondrial sequences of the 13 PCGs. The nodal values indicated the bootstrap value.

## Data Availability

The complete genome sequence of *Polyura narcaeus* has been assigned GenBank accession number MW683125. https://www.ncbi.nlm.nih.gov/nuccore/MW683125. The associated BioProject, SRA, and Bio-Sample numbers are PRJNA716498, SRR14040768, and SAMN18436814 respectively.
